# Inter-observer and intra-observer reliability in the radiographic diagnosis of avascular necrosis of the femoral head following reconstructive hip surgery in children with cerebral palsy

**DOI:** 10.1007/s11832-016-0723-y

**Published:** 2016-03-14

**Authors:** Kim Hesketh, Wudbhav Sankar, Benjamin Joseph, Unni Narayanan, Kishore Mulpuri

**Affiliations:** Department of Orthopaedic Surgery, BC Children’s Hospital, 1D.66, 4480 Oak St, Vancouver, BC V6H 3V4 Canada; The Children’s Hospital of Philadelphia, Philadelphia, PA USA; Paediatric Orthopaedic Service, Manipal, Karnataka India; Hospital for Sick Children, Toronto, ON Canada; Department of Orthopedics, University of British Columbia, Vancouver, BC Canada

**Keywords:** Cerebral palsy, Avascular necrosis, AVN, Inter-observer and intra-observer reliability, Reconstructive hip surgery for children with CP

## Abstract

**Purpose:**

The incidence of avascular necrosis (AVN) following reconstructive hip surgery in cerebral palsy (CP) ranges from 0 to 69 % in the current literature. The purpose of this study was to determine the inter- and intra-observer reliability of radiographically diagnosing AVN in children with CP after hip surgery.

**Methods:**

A retrospective review of 65 children with CP who had reconstructive hip surgery between 2009 and 2012 at BC Children’s Hospital was completed. Anterior–posterior and lateral radiographs were presented to four pediatric orthopaedic surgeons over two rounds. Surgeons were asked to review the set of unidentified radiographs and comment ‘yes’ or ‘no’ for the presence of AVN. Two weeks later the same set of radiographs was sent in a different order and the surgeons were again asked to comment on AVN. Inter- and intra-observer reliability was determined using kappa statistics.

**Results:**

The intra-observer reliability ranged from 0.65 to 0.88 with an average score of 0.76. Inter-observer reliability showed greater variability, ranging from 0.41 to 0.77 with an average score of 0.56 across all surgeons.

**Conclusions:**

Although the intra-rater reliability produced a strength of “good” and the inter-rater reliability a strength of “moderate” agreement, the variability within these scores is clinically important as it demonstrates the difficulty in identifying AVN. This may explain the variability in AVN that is reported in the literature. The need for further education and research in the diagnosis of AVN in children with CP who have undergone reconstructive hip surgery is clinically necessary.

## Introduction

Hip displacement is the lateral migration of the femoral head from the acetabulum and is one of the most common musculoskeletal problems in children with cerebral palsy (CP) [1–4]. It can result in pain, reduced range of motion (ROM), skin breakdown, difficulty with perineal hygiene, and difficulty with seating and positioning, and can contribute to the development of scoliosis and pelvic obliquity [3, 7, 9, 10].

Reconstructive surgery for displaced hips has become the standard of care for children with CP [2, 17, 21]. This typically involves a varus derotational osteotomy (VDRO) of the femur and muscle releases of the adductors and hip flexors. An open reduction and pelvic osteotomy may also be required depending upon the degree of displacement. Goals of the surgical intervention include maintaining or attaining a flexible, aligned, and painless hip [24].

Avascular necrosis (AVN) of the femoral head is a potential complication when undertaking hip surgery in children with CP. The exact pathophysiology is unknown, but may be that the blood supply to the hip is disrupted, leading to cellular necrosis. For children with CP following reconstructive surgery, it is hypothesized that AVN may occur from disturbance in the blood supply to the femoral head during soft tissue releases in the groin, injury to vessels that ascend the femoral head during the VDRO, or excessive pressure on the femoral head when there is insufficient shortening [16, 18]. There is no detailed description of AVN in this population, making it difficult to classify. However, it is well documented in developmental dysplasia of the hip (DDH). Kalamachi and MacEwen is the most common classification in this population [12]. It was first described in 1980 and modified by Kruczynski in 1996 [15]. The severity of AVN in this population can range from mild, involving a hypoplastic epiphysis of the femoral head, to severe, with damage to the growth plate resulting in growth disturbances and misalignment of the proximal femur [19]. The clinical effects of AVN are also not well documented in the CP population. However, idiopathic AVN has been reported to cause pain, limited ROM, and an altered gait pattern [13]. In children with developmental dysplasia of the hip, Roposch et al. reported there is a large variation in the clinical presentation of AVN. Severe forms lead to hip pain and premature debilitating osteoarthritis [19]. Mild forms may have minimal deformity and dysfunction [19]. Pain and the associated clinical relevance of AVN may be underestimated in children with CP due to the inherent difficulty in quantifying pain and symptoms for this challenging patient population.

The incidence of AVN after hip surgery in children with CP varies in the literature from 0 to 69 % [2, 4, 6, 7, 11, 14, 16, 18, 22, 23]. The disparity in the reported rates of AVN may be due to a number of reasons. First, the differences may be related to the variability in risk for AVN development across series. Second, inadequate follow-up of patients may contribute to under- or over-reported AVN. Finally, and most relevant to our study, the disparity in reported rates of AVN may be related to differences in diagnosing and identifying AVN radiographically. To date, there is no known evidence documenting the reliability amongst orthopaedic surgeons in identifying and diagnosing AVN in children with CP following hip surgery. The purpose of this study was to determine the inter- and intra-observer reliability of pediatric orthopaedic surgeons radiographically diagnosing AVN in children with CP after hip surgery.

## Materials and methods

A retrospective review of children, ages 2–18 years, with CP who had reconstructive hip surgery for hip displacement between 2009 and 2012 at BC Children’s Hospital was completed. Patients were excluded if there was no diagnosis of CP or they had undergone salvage surgery. A sample of 65 anterior–posterior and frog-lateral radiographs was selected for the study.

Radiographs were taken by a single radiology department in a standardized fashion and collected at post-surgical follow-up visits. Anterior–posterior and frog-lateral radiographs for each case were presented in random order to four pediatric orthopaedic surgeons over two rounds. The radiographs were saved from an electronic database then pasted and randomized in a Word document for the surgeons to evaluate. In the first round surgeons were asked to independently review the set of unidentified radiographs and comment ‘yes’ or ‘no’ for the presence of AVN. Two weeks later the same set of radiographs was sent in a different order and the surgeons were again asked to comment ‘yes’ or ‘no’ for the presence of AVN. Because the diagnosis of AVN is a highly subjective process and there is no standardized method for objective diagnosis in this population, no defined criteria to assess for AVN was provided. Diagnosis was left to the subjective interpretation of each surgeon, to reflect the current practice for AVN diagnosis. Each surgeon was experienced with the pediatric hip.

The inter- and intra-observer reliability was determined using kappa statistics as described in Altman [1], indicating that agreement <0.2 is poor, 0.21–0.40 is fair, 0.41–0.60 is moderate, 0.61–0.8 is good, and 0.81–1.00 is very good.

## Results

Sixty-five radiographs of both left and right hips were evaluated to give a total of 130 hips. Since each hip was individually classified by the presence or absence of AVN, reliability should not be affected by side other than by chance or bias. Thus, each hip can be considered unique and reliability values are reported across all 130 hips. A secondary analysis was performed to examine right and left hips separately.

### Total AVN diagnosed

**Fig. 1:**
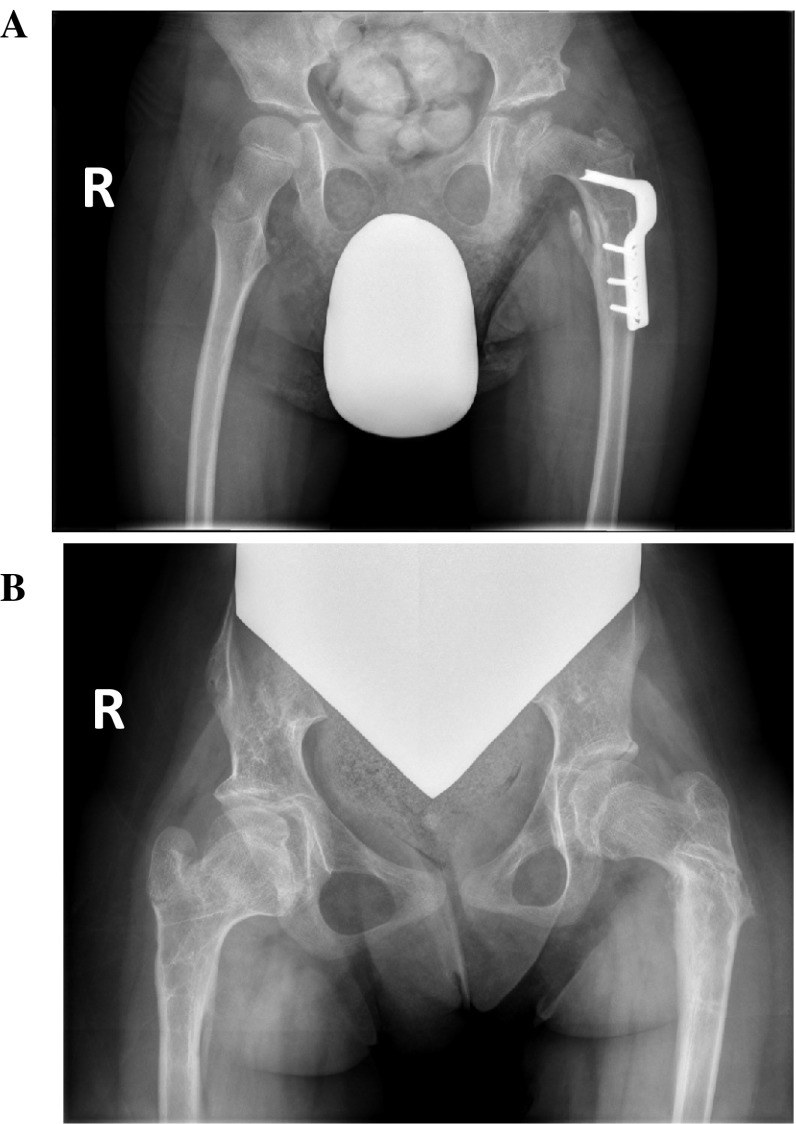
Optimal and suboptimal agreement between pediatric orthopaedic surgeons on the presence of avascular necrosis (AVN) of the femoral head in children with cerebral palsy. Anteroposterior radiographs of the hips were identified to have AVN by **a** 4/4 surgeons (*left hip*), and **b** 2/4 surgeons (*right hip*)

To quantify how many hips in total had AVN, those in which at least two surgeons identified AVN were considered positive. In trial one, 23 hips (18 %; 10 right, 13 left) were identified as having AVN, while in trial two, 21 hips (16 %; 8 right, 13 left) were identified. Of the right hips, seven were identified in both trials, with three and one hips independently identified in trials one and two, respectively. Of the left hips, 11 were identified in both trials with two hips independently identified in each. Between the two trials, a total of 11 right hips and 15 left hips in 25 different patients were diagnosed with AVN (Table 1). There were eight hips in which all four surgeons positively identified AVN, and eight hips in which only two surgeons identified AVN. Example radiographs of these optimal and suboptimal agreements are shown in Fig. 1a, b, respectively. In comparison, there were 81 hips (41 right, 40 left) in which all four surgeons reported no AVN in either trial.

**Table 1:** AVN-positive hips—number of surgeons reporting

Subject no.	No. of surgeons reporting AVN			
	Right hip		Left hip	
	Trial 1	Trial 2	Trial 1	Trial 2
1	–	–	4	4
2	–	–	3	3
3	4	3	3	3
11	–	–	2^a^	1
15	4	4	–	–
17	2^a^	1	–	–
19	–	–	2	2
20	–	–	4	4
21	–	–	2	3
22	–	–	4	4
23	–	–	4	4
27	–	–	1	3^a^
28	4	4	–	–
29	2^a^	1	–	–
30	4	3	–	–
33	3	3	–	–
34	–	–	2	3
35	1	2^a^	–	–
47	–	–	0	3^a^
48	4	4	–	–
49	4	4	–	–
52	–	–	2^a^	1
53	–	–	4	3
54	–	–	2	2
65	2^a^	1	–	–

^a^Indicates hip met AVN criteria for only the specified trial; *dashes* indicate hip was classified as AVN-negative

### Intra-observer reliability

The intra-observer reliability showed variability among the surgeons, ranging from 0.65 to 0.88 with an overall mean score of 0.76 (Table 2). Across all hips, the intra-observer reliability of all four surgeons was relatively high. Surgeon 4 had very good intra-observer reliability (0.88), and surgeons 1, 2, and 3 had good agreement (0.80, 0.72, and 0.65, respectively). Looking at the right and left hips separately, the intra-observer reliability was more variable. Surgeon 4 demonstrated very good intra-observer reliability for both right and left. Surgeon 1 had very good agreement for the right hip and good agreement for the left. Surgeon 2 demonstrated good agreement for both sides, while surgeon 3 had good agreement for the right and moderate agreement for the left (Table 2).

**Table 2:** Intra-observer reliability—kappa values and interpretation

	Kappa values			
	Surgeon 1	Surgeon 2	Surgeon 3	Surgeon 4
All hips	0.80 (G)	0.72 (G)	0.65 (G)	0.88 (V)
Right hip	0.84 (V)	0.69 (G)	0.69 (G)	0.82 (V)
Left hip	0.77 (G)	0.75 (G)	0.60 (M)	0.93 (V)

Altman [1] kappa values: V, very good; G, good; M, moderate

### Inter-observer reliability

The inter-observer reliability was lower and showed greater variability than intra-observer reliability, ranging from 0.41 to 0.77 (Table 3) with an overall mean score of 0.56 (moderate). Surgeon pairs 1–2, 2–3, 2–4 and 3–4 scored moderate across all hips with means of 0.50, 0.47, 0.43, and 0.57, respectively. Surgeon pairs 1–3 and 1–4 scored good across all hips with means of 0.69 and 0.72, respectively Looking at right and left hips separately, inter-observer reliability had a larger variation but a higher mean in the right hip compared to the left (Table 4). Overall means for surgeon pairs for the right and left hip were 0.60 and 0.54, respectively, both a score of moderate.

**Table 3:** Inter-observer reliability––kappa values and interpretation

Surgeon pair	Kappa values		
	Trial 1	Trial 2	Mean
1–2	0.46 (M)	0.55 (M)	0.50 (M)
1–3	0.63 (G)	0.74 (G)	0.69 (G)
1–4	0.77 (G)	0.67 (G)	0.72 (G)
2–3	0.49 (M)	0.46 (M)	0.47 (M)
2–4	0.45 (M)	0.41 (M)	0.43 (M)
3–4	0.62 (G)	0.53 (M)	0.57 (M)

Altman [1] kappa values: *G* good, *M* moderate

**Table 4:** Inter-observer reliability by hip—kappa values and interpretation

Surgeon pair	Kappa values		
	Trial 1	Trial 2	Mean
Right hip			
1–2	0.48 (M)	0.48 (M)	0.48 (M)
1–3	0.74 (G)	0.63 (G)	0.69 (G)
1–4	0.84 (V)	0.82 (V)	0.83 (V)
2–3	0.5 (M)	0.47 (M)	0.49 (M)
2–4	0.37 (F)	0.48 (M)	0.43 (M)
3–4	0.72 (G)	0.63 (G)	0.67 (G)
Left hip			
1–2	0.44 (M)	0.59 (M)	0.52 (M)
1–3	0.53 (M)	0.83 (V)	0.68 (G)
1–4	0.72 (G)	0.57 (M)	0.65 (G)
2–3	0.49 (M)	0.45 (M)	0.47 (M)
2–4	0.54 (M)	0.36 (F)	0.45 (M)
3–4	0.53 (M)	0.43 (M)	0.48 (M)

Altman [1] kappa values: *V* very good, *G* good, *M* moderate, *F* fair

## Discussion

The frequency of AVN in children with CP after reconstructive hip surgery varies in the literature from 0 to 69 % [2, 4, 6, 7, 11, 14, 16, 18, 22, 23]. The diagnosis of AVN is a highly subjective process dependent upon the individual surgeon and there is no standardized, rigorous method for objective diagnosis. One possible reason for the incidence variability in the literature may be the ability to identify and report AVN. The purpose of this study was to determine the inter- and intra-observer reliability of orthopaedic surgeons for the radiographic diagnosis of AVN in children with CP after hip surgery. Although the intra-observer reliability produced a strength of good agreement and the inter-observer reliability produced a strength of moderate agreement, the variability within these scores is clinically important as it demonstrates the difficulty in identifying AVN. These results may account for much of the disparity in the literature in the reported frequency of AVN in children with CP after reconstructive hip surgery.

A study completed by Davidson et al. investigating the reliability of diagnosing AVN in children with slipped capital femoral epiphysis (SCFE) reported very high inter- and intra-observer reliability [5]. The discrepancy when compared to the identification of AVN in children with CP indicates there may be a specific difficulty in diagnosing AVN within the CP population.

There are a few limitations with this study. No standard definition or classification scheme of AVN was given to the surgeons. This reflects how AVN is reported in the current literature; there are no clear guidelines used when identifying and reporting AVN after hip surgery in children with CP. Our study evaluated the reliability amongst experienced surgeons only. Within this small sample of surgeons, those with the most clinical experience had better intra- and inter-observer reliability. Including fellows and residents, who are often involved in identifying post-operative complications, may have led to even greater variability in the identification of AVN. This study was limited to the radiographic diagnosis of AVN and did not include the use of magnetic resonance imaging (MRI). Using the Ficat classification scheme of idiopathic AVN, Schmitt-Sody et al. found an inter-observer mean reliability coefficient of 0.36 for radiographs and 0.37 for MRI [20]. Ficat reported that early signs of AVN are not visible on radiographs [8]. Although MRI offers a more detailed view of the hip, these examinations are not used on a routine basis with our patient population and are therefore less clinically relevant.

To identify risk factors and decrease the incidence of AVN, studies evaluating the outcome of reconstructive hip surgery in children with CP need to investigate and report the incidence of AVN. A classification system to identify and quantify the severity of AVN in this population is also necessary. The results of this study suggest that the reliability of radiographic diagnosis of AVN in children with CP after reconstructive hip surgery is “moderate”. These results may explain the current variability in the frequency of AVN reported in the literature. In order to improve the reliability of the diagnosis of AVN there needs to be a clear definition and classification system used. The need for further education and research in the diagnosis of AVN in children with CP who have undergone reconstructive hip surgery is clinically necessary.
